# Re-enacting the Bodily Self on Stage: Embodied Cognition Meets Psychoanalysis

**DOI:** 10.3389/fpsyg.2019.00492

**Published:** 2019-04-05

**Authors:** Claudia Scorolli

**Affiliations:** Department of Philosophy and Communication Studies, University of Bologna, Bologna, Italy

**Keywords:** psychoanalysis, embodied cognition, psychoanalytic psychodrama, intersubjectivity, sentient bodily self, affects, internet addiction disorders, eating disorders

## Abstract

The embodied approach to cognition consists in a range of theoretical proposals sharing the idea that our concepts are constitutively shaped by the physical and social constraints of our body and environment. Still far from a mutually enriching interplay, in recent years embodied and psychoanalytic approaches are converging on similar constructs as the ones of intersubjectivity, bodily self, and affective quality of verbal communication. Some efforts to cope with the *sentient* subject were already present in classical cognitivism: having expunged desires and conflicts from the *cognitive harmony*, bodily emotions re-emerged but only as a noisy dynamic friction. In contrast, the new, neural, embodied cognitive science with its focus on bodily effects/affects has enabled a dialogue between neuro-cognitive perspectives and clinic-psychological ones, through shared conceptual frameworks. I will address crucial issues that should be faced on this reconciling path. With reference to two kinds of contemporary addictions – internet addiction disorder and eating disorders – I will introduce a possible therapeutic approach that is built upon the core role of the acting-sentient bodily self in a dynamic-social and affective environment. In Psychoanalytic Psychodrama, the spontaneous re-enactment of a past (socially and physically constrained) experience is actualized by means of the other, the Auxiliary Ego. This allows homeostatic and social-emotional affects, i.e., drives and instincts, to be re-experienced by the agent, the Protagonist, in a safe scenario. The director-psychoanalyst smoothly traces back this simulation to the motivated, and constrained, early proximal embodied interactions with significant others, and to the related instinctual conflicting aims. The psychoanalytic reframing of classical psychodrama does not merely exploit its original cathartic function, rather stands out for exploring the interpersonal constitution of the self, through an actual “re-somatization” of psychoanalytic therapy. Unspoken/unspeakable feelings pop up on stage: the strength of this treatment mainly rests on re-establishing the priority of the *embodied Self* over the *narrative Self*. By pointing out the possible conflicts between these two selves, this method can broaden the embodied cognition perspective. The psychodramatic approach will be briefly discussed in light of connectionist models, to finally address linguistic and methodological pivotal issues.

## Introduction

The aim of this work is to prepare the ground for an enriching dialogue between cognitive science and psychoanalysis by identifying common theoretical constructs and potential new methods. In this view, the introduction is dedicated to clarify the specific nature of cognition assumed by the present article, that is, an “embodied” nature. It denies that representations are built out of amodal symbols and claims that sensory, motor, and affective processes are intrinsic to cognition.

The argument is articulated by first discussing recent neuroscience literature. From a “cognitive” to an “affective” neuroscientific perspective, scientists are nowadays converging on themes that traditionally belonged to psychoanalytical approaches as the ones of the bodily self, intersubjectivity, unconscious, and prosodic-affective quality of verbal communication. Along with the common questions, divergences are also addressed and traced back to two scarcely reconcilable objectives. In cognitive science view the objective is to generalize and to predict; in psychoanalysis the objective is to dig deep into the unique self and its conflicting states.

To clarify the convergences, in what follows four thematic cores are addressed (environment; drives, instincts, and unconscious conflicts; intersubjectivity; perception of the body) and are connected with two disorders: internet addiction disorder and eating disorders (EDs). This problematization will allow the introduction of the psychodramatic method and the contrast between an “acting” cure and a “talking” cure. Next, the psychoanalytic reframing of classical psychodrama is introduced: psychoanalytic psychodrama does not merely exploit the cathartic function but stands out for exploring the self in its interpersonal constitution, through an actual somatization of psychoanalytic models, prioritizing the embodied self over the narrative self. Its distinctive features are discussed against traditional psychoanalytical approaches and body-oriented therapies.

To further ease an effective interdisciplinary collaboration, a possible integration with ecological connectionists models (implementing the grounding in sensory, motor, and affective processes as intrinsic to cognition) is outlined. Finally, linguistic and methodological issues are addressed: shared constructs across disciplines are necessary to make effective and substantial advancements within this new science. To encompass the sentient bodily self, by empirically supporting this exploration also outside the therapeutic setting, the further challenge of neuropsyhcoanalysis requires the exploitation, and the integration, of the most promising methodologies. This effort would guarantee the necessary flexibility with respect to different settings-contexts, as well the opportunity to investigate different issues.

## Which Cognitive Science?

Nowadays the adjective “cognitive” is abused, generating a lot of confusion about its meaning. The reason is that there is not a single cognitive science, but at least two. The first one is the classical cognitive science, “Cognitivism”: a computational science according to which the mind, similarly to the software of a computer, is a mechanism for manipulating arbitrary and amodal symbols. This science basically arose from the ashes of behaviorism. Consistently it had a twofold aim: rejecting behaviorism while maintaining scientificity and preserving the “science of mind” separated by neuroscience (Parisi, 2002).

Progress in neuroscientific research, as well as gradual awareness that computing machines lack human-like intelligence, created the demand for a new cognitive science. This science had to consider not only the brain, but also the body (Glenberg, 1997; Barsalou, 1999; Gallese and Lakoff, 2005): this neural cognitive science can be identified with “Embodied Cognition”. Embodied cognition has led to a conceptual revolution by demonstrating that concepts consist in the reactivation of the same neural activation pattern involved when we perceive objects/entities they refer to and when we interact with them (Scorolli and Borghi, 2007; Scorolli et al., 2012; Borghi et al., 2013; Scorolli, 2014). The plethora of recent theories, labels, and approaches traceable within this perspective may appear puzzling: a clear conceptual clarification is provided by Pezzulo et al. (2013). While the label “grounded” refers to the physical foundation of cognition, affected for instance by the laws of physics, the label “embodied” specifically points to the physical constraints of our body, i.e., to the sensory-motor experiences shaping our cognition. Finally, the label “situated” nicely refers to the dependence of cognitive processing on both current constraints and tasks demands. These level-specific modulations exert additive effects on cognition. They can be conceptualized as a cascade, ranging from the physical properties of the world to the sensorimotor, affective and interoceptive processes, and in the end to the specific characteristics of the environment, including its social and cultural features (Pezzulo et al., 2013).

Regardless of the taken perspective, these theoretical proposals share the idea that grounding phenomena are constitutive of cognition, thus are not just optional add-ons. In the present work, the label “embodied” is privileged to directly point to bodily, affective, and emotional states (Niedenthal et al., 2005; Wilson-Mendenhall et al., 2011), though without disregarding the role, functions, and requirements of the physical and social context.

## Embodied Cognition and Psychoanalysis

Previous attempts towards a mutually enriching dialogue between neuroscience and psychoanalysis have been mainly focused on “the new neuropsychological theoretical and methodological apparatus of cognitive neuroscience (that) can be easily and profitably incorporated into theoretical psychoanalysis without any cost” (Semenza, 2014, p. 3). This kind of approach assumes that emotions, feelings, affects, and drives can be first neglected, as a background noise with respect to the investigation of action and perception, or memory and language processes; they can be re-integrated into the cognitive scaffolding at a later stage. The risk is not providing an account for “authenticity, individuality, feeling, and character” (Brown, 2014, p. 10). In contrast, embodied theories claim that mind is not just cognition, intelligence, language, skills, but also motivation, perceived sensations, and felt emotions (i.e., bodily effects). Despite this theoretical claim, only in recent years there has been a growing attention (also) for empirical data pertaining to the emotional and the motivational spheres (thus belatedly with respect to the breakthrough of *affective neuroscience*: Panksepp, 1998, 2005; Damasio, 2000; Damasio et al., 2000). This interest has been fostered by several factors and new scientific challenges:

– The emergence of an embodied *social* cognition, and thus the interest not just in motor automatic resonance mechanisms, but also in complementary (non-imitative) actions involved for instance in joint activity (Rumiati and Bekkering, 2003; Sartori and Betti, 2015; see also the upcoming research issue by Brunel et al., 2019).
– The notable advances in artificial life simulations, that have shifted the attention to populations of organisms that evolve biologically, to their specific needs for survival and reproduction, and hence to the motivations deriving from them (Parisi, 2013, 2014).
– The acknowledgment that psychological phenomena are complex, determined by a huge number of variables interacting with each other non-linearly (e.g., the emergence of mental disorders from the interactions of different symptoms), and thus the necessity to approach them from a complex systems perspective (e.g., Borsboom et al., 2018).
– The novel evidence and theoretical accounts of the representation of abstract concepts, which emphasize the role of emotions and sociality in grounding abstractness (see the recent theme issue by Borghi et al., 2018).
– The new frontier research on interoception, and its central role for cognition, that breaks out of corticocentricity. In the special issue on the impact of interoception beyond the homeostatic/allostatic reflexes (Tsakiris and Critchley, 2016), authors from different perspectives address how information about the internal states of the body are integrated with thoughts, feelings, and behaviors. A compelling body of empirical evidence and neurobiological insights is discussed to explore also clinical implications and methodological issues.
– The final factor impacting current embodied perspectives definitely is the widespread availability of neuroimaging techniques for the investigation of those brain regions that are associated with cognitive-emotional interactions. Both indirect measurements (based on hemodynamic responses) and direct measurements (based on brain electrical dynamics) are making a difference in the examination of the interplay between emotional control and cognitive regulation (Tyng et al., 2017).

Beyond these factors, in last years neuroscientists, philosophers, computer scientists, and roboticists have re-evaluated psychological schools that ruled in Europe in the first half of the twentieth century: Gestalt theory of perception, Piagetian and Vygotskian theories of cognitive development, and Freudian theory of psychosexual development. The theoretical frameworks of these scholars range from affective neuroscience to embodied cognition (e.g., Panksepp and Solms, 2012; Rizzolatti et al., 2014): they share with the psychoanalytic approach the idea that the supremacy of “the cognitive” is made unsettled by “the dynamic”.

Regardless of the attempts of a worthwhile dialogue, cognitivism can hardly be integrated with psychodynamic theorization, which instead appears inherently related to new embodied approaches. With them, it shares at least the following views: (1) The rationality is seen as the tip of the iceberg; (2) the refusal of the primacy of the intellect over the emotions and the primacy of the mind over the body; (3) the “core self” is conceived as the continuous interaction between intero- and exteroceptive stimuli (Northoff, 2012); (4) the individual mind is not simply “brainbound” but also distributed beyond the body’s edges (Hutchins, 1995) across non-bodily devices but also bodily social ones (Clark, 2003; Thompson and Stapleton, 2009; Wilson, 2010) through interpersonal body representations (Costantini et al., 2011); (5) a “genetic epistemology” approach, meaning that we can better understand X if we reconstruct how X has become what it is (Piaget, 1952; Parisi and Schlesinger, 2002): accordingly, our mind cannot be conceived as just extended but, for the immature infant, also in need of boundaries for the overwhelming ambivalent stimuli (Freud, 1920; a skin to the mind: Levy and Lemma, 2004; a containing mind: Bion, 1962); (6) many simultaneous causes produce many effects on the self, in largely unpredictable ways: to grasp the complex interplay of brain, body, and environment, a dynamical system approach is needed (Clark, 1999); (7) the acknowledgment of the limitations of science in predicting effects before they occur, and controlling all variables; and (8) the concerns about the scientific method: not the only way, and perhaps not even the best, for our knowledge of reality.

## Shared Concepts and Objectives but Censorships

### Unconscious, Empathy, and Intersubjectivity

As insightfully reviewed by Kihlstrom (2015), the term “unconscious” was used by Freud with three different meanings: to refer to thoughts, feelings, or desires that are not aware at any particular time; or available to consciousness in principle, even if they are not accessible at the present moment (“preconscious”); or not even available to consciousness. Nevertheless, in the *dynamic* sense of the term, only repressed mental contents were conceived as “unconscious”, like sexual and aggressive impulses prevented from being represented in consciousness (though still determinants of our thoughts and actions). The goal of the psychoanalytic treatment is to bring these contents into consciousness in order to be dealt with.

We can easily guess that within the scientific-academic milieu this construct was not welcomed. While widely popular and evocative among clinicians and the public, the first blow was knocked by the behaviorist revolution. Concerned about raising psychology to the level of science, behaviorism has drastically cut off the unobservable mental life. The following breakthrough of cognitivism led to progressive evidence on implicit perception, like in subliminal priming effects (e.g., Pesciarelli et al., 2019), or on the distinction between automatic and controlled cognitive processes, like in the Stroop effect (e.g., Scorolli et al., 2015). This new evidence in some way legitimized the construct of unconscious to refer to “unconscious cognition” (Kihlstrom, 1987), thus in a descriptive-systematic sense, not in its dynamic foundational meaning. If we define cognition as pertaining to any mental activity, unconscious cognition could not disregard feelings and desires (Kihlstrom, 2013). Nevertheless, the unsolved issue was still related to how, in the absence of conscious awareness, emotions and motivations can affect our thoughts and actions. The “explicit”/“implicit” distinction is more difficult to be dealt with when encompassing the emotional sphere. Some behavioral and/or physiological components of implicit emotions can be objectively measured (though it is challenging to link them to the corresponding “source of stimulation”). Some other components can be caught only through “projective” measurements, dream imagery, and free associations (considering that the same cause can produce different effects, and not all these effects are synchronous). Kihlstrom (2015) foresees a future for the dynamic unconscious in the current renewed interest in consciousness, though recognizing that “carefully controlled experiments (…) have not yielded much evidence favoring the view of the dynamic unconscious as conceived in psychoanalytic theory, with its unconscious conflicts over primitive sexual and aggressive motives” (p. 996). To achieve a substantial breakthrough, it is probably necessary to change the perspective, introducing new concepts as well.

The concept of empathy, neglected by classical cognitive science, has been retrieved by the new neural embodied science. “A path leads from identification by way of imitation to empathy” (Freud, 1921, p. 110): such a farsighted statement has been neuro-physiologically accounted for by embodied cognition. The discovery of mirror neurons, and embodied simulation, has laid the foundation for the neuroscientific study of intersubjectivity and empathy as means of inter-individual transfer of meaning, opening the way to connect Freudian theory of the psyche with the most advanced scientific theories and discoveries (Rizzolatti and Sinigaglia, 2006; Gallese, 2007a).

With regard to the internal world, recently embodied approaches have emphasized the importance of emotions (Caruana and Gallese, 2011, 2012), bodily self (e.g., Ferri et al., 2012), and indirectly of the unconscious as well. Damasio (1999) refers to “nuclear consciousness”, whose foundation does have an emotional nature, thus it does not imply language. This implicit memory of the perceptions of self-with-others, which follow over time during the first year of life, is tacit, non-declarative, procedural, and, similarly to the Freudian unconscious, does have a crucial role in adult social life and relationships. The related “unconscious emotions” seem to be controlled by a subcortical circuit (amygdala, superior colliculus, and pulvinar). The so characterized unconscious does not correspond to Freudian “topical” unconscious (the id in the second topical), and there is no reference to Freudian repression; nevertheless the role of *what is not accessible to consciousness* is acknowledged and emphasized in the psychoanalytic relationship (for a recent reconsideration of the anatomical localization of consciousness with cross-references to Freudian view, see Solms 2017b, 2019). The patient and the psychoanalyst can unconsciously grasp, in a continuous and reciprocal motion, subtle stimuli of the other, that activate shared neural patterns (Gallese et al., 2006; Ginot, 2015).

### Verbal Communication and Its Prosodic-Affective Quality

Unlike psychoanalytic approaches, cognitivist ones seem to suggest first to ignore the affective noise, desires, and conflicts (at intrapersonal and interpersonal/intergroup level) and later to attach them to the impoverished reconstructed mind. Conversely, Freudian and embodied views share striking similarities, for instance, when accounting for speech (Freud, 1915b; Gallese, 2007b, 2008). As masterly reviewed by Gallese (2009), within the analytic relation the affective quality of verbal communication assumes a critical role in terms of prosody (Rizzuto, 2008), rhythm, tone, timbre, and musicality, as well as syntax and tempi of speech (Mancia, 2006; Rizzuto, 2008; Gallese, 2011).

Recent evidence on language acquisition has shown that the ability of 4-day-old infants to distinguish utterances in their native languages from those of another language depends on prosodic cues (Mehler et al., 1998). Gervain and Werker (2013) have shown that in 7-month-old bilinguals (succeeding in mastering their mother tongues as efficiently as monolinguals), the precocious and effortless acquisition of grammar is actually accounted by their skill on exploiting characteristic prosodic cues (pitch and duration). Furthermore, when investigating the development of language-specific trochaic bias in German-French bilinguals (i.e., languages with and without a trochaic lexical stress), Bijeljac-Babic et al. (2016) found that listening preferences of these 6-month-old infants were comparable to those of German-learning monolinguals but differed from those of same age French-learning monolinguals (i.e., no preference: Höhle et al., 2009). This precocious emergence of a trochaic bias even in simultaneous bilinguals further supports the importance of prosodic information. Finally, it seems that precocious language learning processes (9-month-old infants) are specifically enhanced by social interaction, while not requiring long-term listening (Kuhl et al., 2003). Overall, this evidence unveils the intimate link among embodied language, prosody, and intersubjectivity: the human language learning system flexibly adapts to and exploits the linguistic, affective-social environment (for a recent work on emotional word processing also in the domain of social cognition, see Herbert et al., 2018).

### Towards a Unique Total Self

A further contact point between embodied cognition and psychoanalysis can be found in their objectives. While cognitivism was aimed at discovering a supposed “normative averageness,” the new neural science is pointing out the significance of idiosyncratic profiles of cortical activation (Gallese, 2007a), as well as the individual characteristics in light of life experiences and unconscious facets (“Unique Total Self”: Schaefer and Northoff, 2017). Similarly, psychoanalytic approaches underline the uniqueness of the patient and the role of the environment in which she grew up (family), which may have fitted or not her basic personality tendencies, as introversion and extroversion (Rapaggi, 1994; John and Srivastava, 1999).

### Limits

The limits, or rather “censorships,” of the new embodied science consist in not having adequately considered that perception is always accompanied by feelings of pleasure or pain, lust or grief (Panksepp, 1998; Cuccio et al., 2013; Alcaro et al., 2017; Solms, 2017a; Moccia et al., 2018): behavior cannot be explained apart from this Aristotelian interweaving (Lo Piparo, 2003). Actually, an analogous tendency to quit digging into bodily drives, repudiating the centrality of sexuality, characterizes also current developments of psychoanalytic approaches (for a critical discussion, see Green, 1995; Fonagy, 2008; Fotopoulou and Tsakiris, 2017). In spite of numerous studies on psychosomatic medicine (e.g. Lisi et al., 2014), only a few empirical studies have tried to approach the appetitive dimension of sexuality, that is, the libido. Embodied evidence has made much progress on the investigation of how we understand the emotions of others, *but:* how do we understand ours? Where do they come from?

*Sexual drives* (specifically defined by source, aim, and object; Freud, 1915a) and the possible fixation of the libido (Freud, 1905) are basically cut out from contemporary neuroscience, even if they laid the foundation, as well as the absolute novelty, of Freudian theorization. The general censorship is at least puzzling. There are a few enlightening exceptions, for instance from a neuropsychoanalytic perspective (e.g., Solms, 2012; Stoléru, 2014) or in a strictly embodied-grounded view (Papies and Barsalou, 2015; for an ethological approach to the study of the early modulation of sexual preferences, see Enquist et al., 2011). These works raise new challenging questions on desires that do not directly result from physiological deprivation.

While *psychic conflicts* may or may not be conscious, *drives* are not conscious per se: they arise from the interaction between biological and environmental stimuli and are dialectically constructed from both pressures (Dunn, 1993). But psychoanalytical theory has actually provided an account for the conscious phenomenology of sexual desire. Considering the oral stage of psychosexual development (0–18/24 months), the mouth is conceived as the primary erogenous zone; remarkably the mouth is also central in babies’ exploration. The time window ranging from 0 to 24 months corresponds to the Piagetian sensorimotor period (Piaget, 1945, 1952); coherently Piaget emphasized the mouthing behavior (mostly in the second stage of the first period, from 1 to 4 months). Recently, changes in mouthing of objects have been empirically identified exactly in this period: children ≤24 months exhibit the highest frequency of mouthing behavior, while children >24 months exhibit the lowest frequency (Tulve et al., 2002; for an observational study on children up to 3 years old, see Juberg et al., 2001). Mouthing has been accounted for as crucial to proactively expose the gastrointestinal tract to environmental antigens (Fessler and Abrams, 2004). But this is not the whole story. Early mouthing is related to the objects it is applied to: the rigidity of an object affects the frequency and pattern of mouthing (and grasping) behavior, suggesting that this activity is not merely under the control of reflexive mechanisms (Rochat, 1987). The interplay between the normal cognitive development, certifying a growing interest in the world, and the normal affective development, rooted on bodily drives and instincts, is deserving to be systematically addressed, beyond the borders of sectorial scientific disciplines.

## Needs, Desires, and Addictions

### Cyber Presence and Eating Disorders

In recent years, new addictions are spreading: they are generically labeled as internet addiction disorder (IAD). They are in some way peculiar as not related to substance abuse, even if they could be still explained by evolutionary models, by referring to the emotional systems of reward seeking and separation distress (Panksepp et al., 2002). There is a debate on whether the last version of the Diagnostic and Statistical Manual of Mental Disorders (5th ed.; DSM-5; American Psychiatric Association, 2013) should have classified these disorders as “mental disorders”. Currently, they are variously assessed: dependency on virtual relationships, cognitive overload, virtual sex addiction, and online gaming. Young (1998) identified three main stages in IAD progress: involvement, characterized by initial curiosity; substitution, when the activities that previously were central do not matter anymore; escape, when more frequent and longer time periods are spent using a PC or a smartphone. To date, the most reliable and updated assessment instrument is the Smartphone Addiction Scale (SAS, Kwon et al., 2013). Diagnoses associated with IAD are anxiety, mood disorders, and impulse control disorders. IAD occurs more frequently in individuals with low self-esteem, social difficulties, notable interpersonal sensitivity, obsessive thinking modalities, compulsive behaviors and/or personalities tending to social withdrawal (Cash et al., 2012; for a theoretical framework on molecular underpinnings of IAD, see Montag et al., 2016).

IAD can be particularly disturbing in adolescents and it is basically related to social aspects: it can be better labeled as “cyber-presence.” Studying 1,500 adolescents, between 14 and 24 years old, the Royal Society for Public Health (2017) has identified the following symptoms: anxiety and depressive states, lack of sleep, obsession related to how appearing, cyberbullying, and a new phenomenon, the “fear of missing out”. The central issues shared by embodied and psychodynamic approaches suggest four possible thematic cores:

– *Environment*: IAD is a post-modernity disease (Valleur and Matysiak, 2004) characterized by a multiform group of disorders in which the object is a lawful and socially accepted-encouraged activity. Both the constant connection and the continuous social pressure towards certain models push to conform to an ideal that is not only aesthetic but also emotional: the specific environment can affect the spread of IAD.
– *Drives, instincts, and unconscious conflicts*: immediate gratification is allowed by the continuous accessibility of the network; in the long term this gratification is often accompanied by subsequent negative effects (Marlatt et al., 1988; Lee et al., 2008; for the assessment see Hoerger et al., 2011). It is interesting that elements of orality (analogously with the oral stage fixation present in some EDs) can be identified in IAD: they result from the ability of the eyes to ingest the images more easily than the mouth can do with food (Rapaggi, personal communication).
– *Intersubjectivity*: the strongest component of the “selfie” (i.e., a photograph that one takes of herself, typically taken with a smartphone and shared *via* social media) is its inner contradiction. What might seem a clear act of narcissism hides strong insecurity and deep anxiety of reassurance, which apparently can only be satisfied by others’ approval, the “likes”. Nevertheless, far from calming the neurosis, publishing a selfie only amplifies it (Diefenbach and Christoforakos, 2017). These adolescents are characterized by a loss of interest for actual friendship and/or affective offline sharing; thus, the social network is no longer an instrument, but it appears as the final gratification goal.
– *Perception of the body*: the social network allows a high (illusory) level of control over one’s body image (e.g., on Instagram, the body becomes the one photographed and shared), as well as over emotions and relational contexts to be shown to “online others”. The selection and photoshopping of the bodily image allow to obtain a broad consensus: the success is quantifiable in the number of “likes”.

The *environment* and the (not) felt body, as well as bodily control (the repression of *drives* and *unconscious conflicts*), do have a crucial role also in EDs. These disorders, although well known, in recent years have reached a transversal distribution: they no longer involve only young women. In anorexia nervosa (AN), the control over the (weight of) own body is realized through stoic restrictions in the quantity (and often the variety) of ingested food. Success is measured through the balance needle and the visual comparison with the mirror. As IAD, AN is also characterized by *interpersonal* social difficulties (Cardi et al., 2018), *body misperception* (Linardon et al., 2018), and problematic sexual functioning (Castellini et al., 2012). Hypothesizing that AN is associated with reduced perceived pleasantness during social interactions, Crucianelli et al. (2016) examined the perception of interpersonal affective touch: tactile optimal touch (3 cm/s) elicited significantly lower pleasantness in patients than in healthy controls. Remarkably, cultural and social factors, that is, the *environment*, can affect the variability of EDs symptoms and their diffusion (e.g., orthorexia, a new disorder more common among the male than female population). As to possible relations between IAD and EDs, in a recent work Rodgers et al. (2013) found that for both males and females the avoidance of body image is associated with social network addiction: for females, IAD and avoidance of body image are predictors of EDs.

## Why Adopt Psychoanalytic Psychodrama?

### The Body I Show, The Body I Feed

In both IAD and EDs, the body is not lived-felt, but rather it is conceived as an instrument: a shape to be photoshopped (IAD) or to be weighted (EDs), in any case a thing to be “fixed” before delivering it to the (virtual) others (IAD), or to the mirror (EDs). In this painful game, there is no space for the actual others and the actual relationships. While behavioral-cognitivist therapies cope with these disorders by focusing on the symptom, the psychoanalytic treatment traces back to the symptom’s affective matrix. Consistently with embodied approaches, it does not conceive the body in an objectifying dimension, but as “the body that I am”, thus rejecting the Cartesian dualism mind-body (Merleau-Ponty, 1945; Husserl, 1952). The body is seen in its entirety: not only as a perceptual and motor system, but also as a set of needs and desires (often to be unmasked). Thus, the body is present in every stage of the psychoanalytic path: for example, during the diagnosis the body is interrogated to trace the root of the symptom and/or its symbolic meaning. Triggering and perpetuating factors of EDs are often related to the relationship with one’s own body and sexuality: an objectified body in which the subject does not recognize herself, thus a body that she avoids. It is no coincidence that EDs, such as IAD, though transversal, have great incidence in adolescence, when the body quickly changes and the internal pressures-drives are as pressing as they are unknown. Synchronic and diachronic aspects of the self are crucial to understand these disorders’ symptoms in light of the attachment functioning, from infancy to adolescence (Amianto et al., 2016). Actually many other “contemporary obsessions” revolve around the body and the (fictitious) need for its control. Consistently, in last years professional roles promising to teach how to exercise the best control over the body are exponentially spreading (e.g., beauticians, personal trainers, and nutritionists). Of course, the bodily self, encompassing instinctual, social and affective components, cannot be grasped by these approaches.

### Language in the Talking versus the Acting Cure

For psychoanalysis language is fundamental: the talking cure is enabled by intentional and affective attunement established within the analytic setting through the flow of words (Gallese et al., 2007; see also Cimatti, 2016). This affective attunement closely resembles how mother and baby perceive each other’s emotional state (Emde, 1988a,b; for an overview on psychoanalytic infant research, see Beebe, 2018).

Embodied evidence has shown how language understanding activates motor simulations, but recent theoretical proposals have also highlighted how words are not only the “recording” of our past experiences, but they can be conceived also as instruments that allow us to perform actions in the external world (Borghi et al., 2013; Scorolli et al., 2016; Borghi et al., 2017). Moreover, through internalized language we can speak to ourselves, supporting our thought processes (Borghi et al., 2017). If words are (social) tools, within a clinical setting it is legitimate to expect possible conflicts between the *self instantiated by language*, in its symbolic, reflective, and narrative aspects, and the *bodily self*, emerging from interpersonal relations (Fotopoulou and Tsakiris, 2017). Words seem to originate from communicative gestures (Corballis, 2002; Arbib, 2005), but during the development (i.e., in the course of a progressive abstraction-symbolization process), they can go far away from authentic-spontaneous actions, becoming the most perfect tool of rationalization and intellectualization (i.e., defense mechanisms). Thus a tool that allows combining symbols/events in a logical way but that fails when dealing with unconscious conflicts, the “noisy frictions.” Frictions that the logic and the hic et nunc boundaries cannot grasp.

### The Original Method and Its Analytic Reframing

For these reasons, an action method seems more suitable to shed new light on the unique total self. *Psychodrama*, conceived and developed by Moreno (1937, 1946, 1951; see also Apter, 2003), employs guided dramatic action to examine various issues raised by the individual (the *Protagonist*). The techniques used are intrinsically social and affective; the key ones are: *doubling, role reversal, mirroring, soliloquy, and symbolized role playing* (for details, see the section on Specificities of Psychoanalytic Psychodrama). Through the action on stage, psychodrama enables past, present, and future life events to be explored. Problems and their possible solutions are enacted rather than just talked about.

Psychodrama, unlike classical psychoanalytic therapy, gives space to the body in action, together with the acting bodies of the others. The *Auxiliary Egos* are participants from the audience taking part to the play in order to enact the specific scene. Psychodrama’s powerfulness has to be found in the spontaneous emergence of possible (unconscious) conflicts between verbal and bodily messages. The glaring example is the one of a patient playing on stage and saying to the chosen auxiliary ego “I love you” while stepping back, thus expressing proximity by words, but acting a distance through the body. When the word is “detached” from the body, psychodrama allows the patient to feel this distance, which cannot be effectively grasped in the talking cure. This distance can be brought to consciousness and even measured on stage. The “talking phase” comes after the drama, i.e., offstage: in this final phase, conscious logical thinking is gradually re-integrated. This phase, the *interpretation* proper, characterizes *Psychoanalytic Psychodrama* with respect to its classical counterpart. Psychoanalytic psychodrama had different definitions across years (see Lemoine and Lemoine, 1972; Jeammet and Kestemberg, 1987). The form to which I am referring to is the therapeutic approach suitable for dealing with the core role of the acting-sentient bodily self in a dynamic-social and affective environment. It is characterized by the spontaneous re-enactment of a socially and physically constrained experience actualized by means of auxiliary egos, in a fixed, intimate and trusted group.

Psychodramatic action can portray events already happened, but also reconstructed dreams, fantasies, or future events (planned or just imagined). The play unveils as a real event, allowing homeostatic and emotional-social affects (i.e., drives and instincts: id’s signature; Solms, 2017a) to be re-experienced by the protagonist in a safe scenario. The core role of the director-psychoanalyst consists in smoothly tracing back the current simulation to the motivated, and constrained, early proximities and interactions. Early interpersonal relations with significant others account for current ones: such early experiences shaped “the constitution of the minimal self, including the progressive sophistication of mental distinctions between ‘subject-object,’ ‘self-other’ and even ‘pleasure-pain’” (Fotopoulou and Tsakiris, 2017, p. 7).

Differently from classical psychodrama, this knowledge method does not have a merely cathartic function. Even if using similar action techniques, it stands out for three main reasons: (a) acted loops (repetition compulsion) are traced back to the source (i.e., the relationships with significant others) directly on stage; (b) instead of listening to words at the expense of the body (i.e., reflective and narrative self), psychoanalytic psychodrama encourages embodied interactions in a given environment, as well as social and affective touch (i.e., proximal intersubjectivity and shared interoception: Fotopoulou and Tsakiris, 2017; von Mohr et al., 2017); (c) at the end of the acted drama, the psychoanalyst (linguistically) delivers the interpretation suggested by the previous action, through retrieving embedded words. Thus, words “follow” the body, rather than constraining its intimate-relational-affective dimension: “the mind is there to serve the body’s needs in a given environment” Fotopoulou and Tsakiris, 2017, p. 9). Importantly, the psychoanalyst never provides “instructions,” neither answers: the point is not replacing the old-wrong behavior with a new-right one, but bringing to consciousness the affective, and adaptive, meaning of the current behavior.

To instantiate the converging issues of embodied and psychodynamic approaches, four thematic cores have been addressed with respect to novel and widespread disorders (IAD), in parallel with already well-known disorders which are taking different context-specific facets (EDs). For the sake of completeness, it is worth noting that several other suffering conditions can benefit from psychoanalytic psychodrama treatment, for example, mood disorders, depression, anxiety, psychosomatic disorders (Dayton, 2006), as well as post-traumatic stress disorders, and grief issues, associated with addiction or related to traumas triggered by many causes (ranging from divorce to death of a loved one, Dayton, 2005).

### Embodied Simulation: Traditional Psychoanalysis and Psychoanalytic Psychodrama

As outlined in the sections dealing with unconscious and verbal communication, traditional psychoanalytic approaches already foresee convergences with “embodied” constructs. Below I further address the connections between psychodynamic and embodied cognition referring to the mechanism of embodied simulation, to finally highlight the intrinsic relation of psychoanalytic psychodrama with embodied theories. The neurophysiological foundation for simulation theories was provided by the discovery of the mirror neurons (di Pellegrino et al., 1992). In order to be visually triggered, these neurons require an interaction between the agent of the action and the object of it (Gallese et al., 2006), thus forming a system for matching observation and execution of motor actions. The homology between macaque monkey F5 neurons and human Broca’s region suggested that the development of the lateral verbal communication system in humans derives from a more ancient communication system based on recognition of hand and face gestures (Rizzolatti et al., 1996; see also Gallese et al., 2004). Consistently mirroring phenomena have also been shown in relation to semantic aspects of language: the same motor areas are recruited when a person is understanding action sentences or actually performing the action (e.g., Buccino et al., 2005; Borghi and Scorolli, 2009; Scorolli et al., 2009). Importantly, evidence of parallel neural responses in speaker and listener talking does have crucial implications for psychoanalysis, since it unfolds through verbal communication, which conceivably constitutes the basis for unconscious communication (Gallese, 2009).

A substantial impact on the scrutiny of the neural underpinnings of social behavior comes from findings showing the critical role of internally generated somatosensory representations in recognizing the other’s emotional state conveyed by facial expression (Adolphs et al., 2000; Adolphs, 2003). Mirroring mechanisms and emotional resonance have been variously underlined across different psychoanalytic concepts: projective identification (Klein, 1946), emotional attunement (Stern, 1985), empathic understanding (Kohut, 2010), mirror role of the mother (Winnicott, 1971), and containing “digesting” mind (Bion, 1962). Nonetheless in classical psychoanalysis the interpersonal emotional exchange between patient and therapist basically occurs through verbal communication, thus in principle it cannot exploit visually conveyed messages. The psychoanalyst resonates to the emotions linguistically expressed by the patient. In a recent work attempting to integrate cognitive behavioral theories, embodied simulations, and psychoanalysis for the treatment of post-traumatic stress disorder, Peri et al. (2015) emphasized the importance of having a therapist present in face-to-face contact.

To sum up, embodied simulation does undoubtedly have crucial implications for classical psychoanalytic approaches: Gallese et al. (2007) even suggested possible neural underpinnings of different psychoanalytic concepts, as the ones of unconscious communication, projective identification, empathic understanding, and therapeutic process. However, the idea that the present work intends to propose is that the psychoanalytic psychodrama, compared to the most classical psychodynamic approaches, allows to better emphasize the common ground between embodied cognition and psychoanalysis, by means of the spontaneous re-enactment of the bodily self in the effective intersubjective interaction with the auxiliary egos. The dramatization facilitates the shifting of the focus on somatic sensations through the first-person experience to be touched, intimately related to the interpersonal dimension (Gallese, 2003, 2005; Keysers et al., 2004; Blakemore et al., 2005; Ebisch et al., 2016). For instance, the possibility to re-experience our own tactile sensations, and to experience the ones of the other (through the *role reversal*), allows to reflect upon bodily conveyed messages that we are not always aware of. Furthermore, re-enacting a scene in a safe-controlled environment allows to suspend acting for pauses and *soliloquies*. Paced by the therapist, soliloquy allows the patient to be led to greater sensitivity to bodily interoceptive signals (Critchley and Garfinkel, 2017), to eventually analyze the responses to one’s own/other’s sensations (e.g., fight or flight, or freezing reactions), disentangling their spontaneous components from the socially learned ones. Crucial in this respect is the evidence of automatic embodiment in the observer’s motor system of the sensory qualities of others’ pain (Avenanti et al., 2005). Another peculiar aspect of the psychodramatic method is the way in which it deals with *projection*, i.e., the archaic and primitive defense mechanism that consists in moving feelings, characteristics, or parts of oneself onto other objects or people. The stage and the auxiliary egos provide a different physical and social context from the one to which the person is accustomed. Acting/re-acting in a novel environment is less constrained by previous associations: this fosters the occurence of spontaneous responses and re-emerging of deep (unconditioned) desires and motivations. Projection mechanisms are progressively unmasked. Finally, psychoanalytic psychodrama allows the subject to really experience, in a protected setting, how much her own slight changes in responding to the same requests can determine an effective change of the current situation. The chronical repetition of events often depends on acquired dysfunctional responses to social environment, as well as on erroneous attributions of desires, motivations, and intentions to others. The repetition compulsion is characterized by circumstances that seem to repeat themselves, over and over again, *beyond subjective agency*: the acting cure seems more effective than the talking cure in dealing with it. In the acting cure, the new adaptation emerges spontaneously, disanchoring the individual from her acquired and rationalized responses. The sensory-motor and affective experience on stage provides the patient with a tangible evidence on the potentiality of her action, that is on the efficacy and persistence of her agency.

### Body-Oriented Therapies and Psychoanalytic Psychodrama

Before addressing the specificities of psychoanalytic psychodrama, along with its original techniques, it is worth examining other body-oriented therapies. Indeed, from a clinical point of view the idea that the body can record, recall, and fix what a disembodied mind represses and loses is not completely original. Other psychoanalytic and non-psychoanalytic methods call upon the body. Let us think to Gestalt therapy or to the various body-oriented psychotherapies. Psychoanalytic psychodrama is not “superior” to these other treatments, not even in contrast to them, although distinguishable by its specificities.

The concept of creative adaptation aligns the theoretical elaborations of Perls (1969) and Moreno (1946; Moreno and Moreno, 1969). Gestalt psychotherapy emphasizes the balance between adaptation and creativity, typical of spontaneous contact processes. Psychodrama underlines the different roles that constitute the individual, to make them less rigid, more suitable and effective in relation to the context, in order to progressively detach them from the influences of significant others. Therefore, both models share the final purpose of enhancing the possibilities of adaptation to the environment. This commonality of objectives derives from the fact that both Moreno and Perls move from a relational anthropological perspective: the development of the individual depends on how she interacts with her living context. While Moreno believes that the self emerges from the roles and that the crystallization of them determines a loss of spontaneity and the inability of creative adaptations, for Perls the psychic functioning is determined by the type of relationship that the individual has with her internal and external environment. Consistently a Gestalt technique is the one of the “empty chair”: the client sits next to the therapist, in front of an empty chair. She can project on the chair a person of real life, an emotional aspect of the self, or even an imaginary character. Thus, she can talk to whom is not in front of her eyes, but present as a fantasy: an internal representation. Entering into the relationship (i.e., the actual experience of feeling) should transform the individual’s internal perception of traumatically distorted relationships, to lastly get in touch with her actual needs. As in the case of psychodrama, the therapeutic intervention does not aim to modify the external situation but rather to reshape its misrepresentation. If projecting emotions onto an empty chair is certainly effective, the actual interaction with another sentient self (in the psychodramatic form, the auxiliary ego), which in turn reacts spontaneously to our verbal and bodily messages, greatly improves the intensity and extent of the exploration, and the powerfulness of the experience (Yablonsky, 1976). Indeed, Gestalt therapy often uses elements similar to the ones of psychodrama (e.g., role reversal), but with a critical difference: in Gestalt clinical approaches, the roles are all played by the patient (or, in the role reversal, by the empty chair), not by other people. Since these roles are all conceived only as projections of the self, there is no need for someone else to actually be there; but this choice refrains from exposing the individual to the unpredictability of the other. Finally, in Gestalt therapy, the group can only identify itself as an observer, never as a participant, while in psychodrama the members of the group experience a deep sense of involvement by interpreting auxiliary and double egos (Rapaggi, 2008).

Among the body-oriented psychotherapy, we could also include other forms of therapies: bioenergetic analysis, revolving around the Reichian idea of “character armor” (muscular tensions reflecting repressed emotion: Reich, 1945; Lowen, 1975); psychosomatic practice, with its holistic approach to patient management, encompassing psychosocial factors (Fava et al., 2017); different kinds of group psychotherapy (e.g., Schermer, 2010); massage therapy, gaining popularity as complementary to medical treatment (Moyer et al., 2004); mindfulness, aimed at reducing cognitive vulnerability to stress and emotional distress (Bishop et al., 2004; Bedford, 2012); and new clinical approaches that integrate bodily and psychological aspects as, for instance, neo-functionalism (Ottoboni, 2013). While acknowledging the important clinical value and theoretical interest of these approaches, psychoanalytic psychodrama differs from all of them not only for its extreme flexibility but also for the following distinctive aspects: the individual on stage is permanently an *active* subject; on stage, the subject does have the opportunity of *directly* experiencing that the change is achieved through a shift in physical-psychological perspective; her own actions’ reshaping allows to break through immutable dynamics. Finally, on stage the physical-tangible *symptom* falls into the background, to be then called back in the analysis of its symbolic *meaning* and its “*usefulness*” in legitimizing the suffering of the patient (Jung, 1938). It is worth noting that this relationally oriented approach not only fosters a full mind-body integration but allows the patient to resolve the past and to visit *her future*. Conceptualizing, planning for, and moving towards the future in a realistic manner can be critical for some suffering conditions as, for instance, post-traumatic stress disorders (Dayton, 2006).

### Specificities of Psychoanalytic Psychodrama

After clarifying to what extent more traditional psychoanalytic theorizations already enable to grasp the connection between psychodynamic and embodied cognition, below I summarize the specificities of the psychoanalytic psychodrama with respect to embodied cognition and classical talking cure:

– *Somatization*. Despite the contemporary general “de-somatization” of psychoanalytic models (Fonagy, 2008), psychoanalytic treatment should actually remain a path throughout the history of the subject’s life, back to early infancy (Bazan, 2018), i.e., a stage in which drives and instincts cannot be represented/encoded/conveyed/unveiled by language. The talking cure inevitably shifts the attention on symbolic, “meaning-based”, mentalistic components of the self (Fotopoulou and Tsakiris, 2017), leaving the body “in the waiting room of the therapist’s office” (Conger, 1994, p. 211). Even when keeping out the components of warmth and intimacy that characterize the relationships with caregivers (“attachment”; Bowlby, 1969), action methods prioritize the rooting and structuring of the self in embodied and enacted experiences. At a very basic level, when the protagonist has to place different auxiliary egos on stage, deciding the specific distance one’s from each other, she re-experiences physical proximity and brings her attention to the social messages conveyed by this distance. This experience should affect also the sense of body: body boundaries are not only flexibly modified by tool use (Tessari et al., 2010), but they are also affected by social interacting, as suggested by the analysis of IAD and EDs.
– *Embodied vs. narrative self*. Embodied simulations occur when we process language, when we perceive a visual scene, and even when we imagine doing or perceiving something, since both real action and mental motor imagery activate a common network of cortical and subcortical motor centers (for an analysis on embodied narratology, see Wojciehowski and Gallese, 2011). Evidence has also shown that the simulation shaped during language processing reflects the real interrelations between our body and external referents, suggesting that the simulation triggered by language is quite precise (Ambrosini et al., 2012). The analyst’s full listening can benefit from the resulting empathy. Nevertheless, if we create our own experience through our actions, if what we experience is shaped by how we act (enactive view, Varela et al., 1991; Noë, 2004), attention should be first paid to the embodied self. By using specific action techniques, and/or restructuring the scene (in a more or less symbolized way), within psychodrama the sentient subject gradually emerges. Unspoken/unspeakable feelings pop up on stage. Interestingly silence is also taken into account. In contrast to silences that indicate awkwardness or distraction, “connectional silence” points to reflective suspensions or to mixed and ambiguous emotions. While clinical research is exploring pauses within speaking turns (Bartels et al., 2016; Durieux et al., 2018; Hill et al., 2018; Visser et al., 2019), in the embodied view not enough attention has been paid to communicative silence, which can convey social intentions and requests.
– *Rationalized words vs. spontaneous body.* Evidence on embodied simulation triggered by observing others challenged the notion that interpersonal understanding consists solely of our explicitly attributing propositional attitudes to others. Indeed, embodied simulations create (within the observer) internal non-linguistic representations of the body states associated with actions, emotions, and sensations (embodied empathic inference, e.g., Avenanti et al., 2005). Consistently when the psychoanalyst (as well as the auxiliary ego) observes the play, she maps the protagonist’s actions onto her own motor system and the protagonist’s emotions onto her own visceromotor and somatosensory systems (Gallese and Sinigaglia, 2011).

However, in the psychodramatic scene, the observer is also a listener. Embodied proposals have suggested to conceive words also in their social and public aspects (i.e., instruments for action) and speaking as performing actions in coordination with someone else (Clark, 1996; Borghi et al., 2013; Scorolli et al., 2016; Borghi et al., 2017). Interestingly, the instrument “word” can detach from the body. It happens because the sentient individual is not “a cognitive harmony”: homeostatic and social affects can conflict with our internalized moral standards and ideals. That is, the bodily self can conflict with the self emerging from reasonable prohibitions and rational aspirations (i.e., the Freudian superego), which are mainly instantiated by language. The phenomenological counterpart of the inner dynamic friction is the action/word conflict. The meaning conveyed by language can conflict with the one conveyed by action, as it dramatically emerges on stage (e.g., the verbally conveyed message “I do not trust you” while physically approaching the auxiliary ego, thus acting a proximity despite the distance expressed by the words). If language is a tool, probably it is also the best tool for intellectualization and rationalization (as to defense mechanisms, see Freud, 1936), i.e., a tool at the service of the reflective and narrative aspects of the self. When on stage (but not necessarily on the psychoanalytic couch), if forced to choose between two conflicting messages, we choose the one conveyed by the “unrationalized–unrationalizable” bodily self.

– *Role reversal*. The protagonist becomes aware of her actual-effective messages mainly through a powerful technique of psychodrama: role reversal (Yablonsky, 1976; Blatner and Blatner, 1988; Holmes and Karp, 1990). That is, the auxiliary ego roles into the protagonist position, re-enacting her actions, and verbal messages. Symmetrically, the protagonist roles into the significant other position and enacts that role, experiencing possible inconsistencies between the mentalized messages and the bodily conveyed ones. Thus, the protagonist looks at herself not just as if in a mirror: the role reversal favors the unveiling of protagonist’s intrapersonal conflicts. Furthermore, this technique allows the protagonist to gain an in-depth understanding of the other, of her intimate affects together with her implicit social pressure (let us think about a student rolling into the teacher position). Embodied evidence has demonstrated the motor effects of changing physical, social, and linguistic perspective (Gianelli et al., 2013): psychodrama shows also the affective effects of preventing the individual from being stuck in her own viewpoint (for a review on the interplay between spatial and social spheres, see Proulx et al., 2016). This technique favors transcending the limitations of egocentricity and prevents the protagonist from being trapped in her own defenses. During the action on stage, also the observing audience gets a deeper understanding of the human dynamic interactions.

For the sake of brevity, I will not address other techniques, such as mirroring (the protagonist looks at the scene re-enacted only by auxiliary egos, thus without participating in the scene, but observing herself from outside), soliloquy (the protagonist expresses her thoughts and feelings aloud), and symbolized role playing, in which the director chooses an object, or an auxiliary ego, to portray a specific emotion/symptom/addiction (e.g., an addition can be portrayed by a tug-of-war with the auxiliary ego; see Rapaggi, 2008). Future events can be enacted too, still in a constrained environment: this further differentiates psychodrama from pure imaginative techniques, drawing it near to virtual reality methodologies (e.g., Parsons et al., 2017).

– *Doubling*. In the technique of doubling, a person from the audience goes into the scene, puts a hand on the shoulder of the protagonist, and expresses a message in her shoes. The double gives voice to the unspeakable emotions of the protagonist. (The auxiliary egos cannot double the protagonist as they are already enacting an affective role in the scene.) She can also perform what the protagonist is unable to do (e.g., expressing anger physically). By touching the protagonist’s shoulder, the double establishes physical proximity and contact, as an extension of the protagonist’s body-mind. The double ego attempts to make conscious unexpressed materials. Both her verbal and physical intervention are often disowned by the protagonist: this resistance is discussed in the interpretative final phase. In the “choral doubling” all the people from the audience, one by one, double the protagonist, prompting more than one view. The evoked feelings and/or creative solutions allow an emotional and cognitive restructuring of the scene.

The double, and the members of the group as well, must refrain from both judging behaviors and suggesting interpretations. When the scene is over, the audience can share evoked events/feelings. Only the psychoanalyst is entitled to suggest hypotheses on unconscious conflicts (for a recent discussion of interpretation in psychoanalysis, see Kernberg, 2016). Remarkably, transference is better handled through the psychodramatic techniques. Symmetrically, the analysis of countertransference (i.e., the analyst’s affective reaction activated by the patient’s materials) takes advantages of the right emotional distance allowed by the continuous distributed reallocation of engaging emotions. Still maintaining the necessary affective resonance, this setting outdistances the analyst from taking part in the patient’s internal conflicts and from influencing the patient with her own value systems (“technical neutrality”).

The points sketched above suggest that psychoanalytic psychodrama can reconcile embodied and psychoanalytic approaches through the spontaneous actual re-enactment of significant relationships. In this sense, this method represents a turning point with respect to traditional verbal psychoanalysis: by prioritizing early reciprocal interactions and emotional engagements, it allows to explore the original interpersonal constitution of the self, by actually conceiving the self as rooted in and structured by embodied and enacted experiences (Fotopoulou and Tsakiris, 2017). For instance, as far as IAD and EDs are concerned, this method *immediately* leads the attention to “the body that I am”, thus on muscles tensions, somatic resistance (Lowen, 1969), homeostatic affects (drives) and emotional social affects (instincts) (Wright and Panksepp, 2012; Solms, 2017a,b). The other on stage allows to re-experience recursive loops in a safe setting and through different perspectives, coming back into contact with authentic needs. A mask can still be used (cf. photoshopped picture in IAD), but then it is removed. The only mirror on stage is the other (cf. EDs and AN), which gives back to the protagonist a complex image of her lived/living body. The gradual re-structuring of the scene allows an actual nutritive experience. Differently from the cyber space (cf. IAD), all the emotions are accepted and not censored (e.g., not only joy but also sadness). Finally, the word (rationality), abused in social networks, is downgraded: if it conflicts with the action, action is what the person trusts.

## Possible Integration with Ecological Connectionist Models and Robotics

The psychodramatic approach is not far from ecological connectionist approaches. These relatively new proposals conceive the neural network as a model of the nervous system of an organism living in a certain environment. This environment is both natural and social, as it contains other organisms with which the (artificial) organism interacts (Cangelosi et al., 2010). In these distributed models a psychological entity (e.g., a concept, a word, a perceived object or its property) is not represented by a single unit, as in localist models, but by a particular pattern of activation of a set of units, thus different entities can be represented by different patterns of activation of the same units. Psychotherapy can be conceived as a sensorial input to the neural network, which, unlike psychotropic drugs, acts in a specific way and does have different effects depending on the specific individual. The current weights of the network can be conceived as the result of all past experiences and learning: the treatment should remove the root causes of the patient’s discomfort by modifying a specific set of weights. Resulting changes in “patient’s network” should be slower but long lasting (Parisi, 1989). In the case of an action method, the input would be not only verbal but also motoric, thus enhancing accumulation of experience.

Interestingly, simulations of a certain learning with neural networks allow to investigate the stages through which that learning is achieved, without the need to postulate the existence of rules (Rumelhart and McClelland, 1986; Plunkett and Marchmann, 1991). In contrast to cognitive-behavioral view, the change (the new adaptive behavior) would not be subordinate to the discovery and the incorporation of rules. With regard to the ecological approach, further possible advantages can be identified in the adoption of an action-based group therapy: the organism is examined in conjunction with its natural and social environment. In a similar vein, recent work in robotics underlines the need for new robots to be equipped with mechanisms of neural and perceptual readiness (Cangelosi and Schlesinger, 2015) but also with “affects” mediating and regulating the sensorimotor behaviors (Zhong et al., 2016). Testing these robots would allow a breakthrough on developmental models, providing crucial hints on the developmental stages of affective subjectivity, to finally scrutinize the interplay between the adaptive brain, the growing body, the responsive social context, and the physical environment (Belpaeme et al., 2016).

## Conclusions and Outlook

Traditionally far away from each other, in very last years the “poorly evidence-based” psychoanalysis and the “censored” cognitive neuroscience are making a common effort towards a possible dialogue. On the cognitive-neuroscientific side, embodied cognition seems inherently suitable to achieve this fruitful and effective dialogue as it lays its foundations on concepts intrinsically shared with psychoanalytic approaches.

Across the present work, these shared constructs are sketched in relation to recent contemporary disorders, IAD and EDs, to finally introduce a therapeutic method suitable to approach the acting-sentient bodily self in a dynamic-social and affective environment. Psychoanalytic psychodrama copes with the spontaneous re-enactment of an actual (socially and physically constrained) experience, actualized by means of auxiliary egos. Drives and instincts can be re-experienced by the protagonist in a safe scenario. The psychotherapist is a “listener” and an “observer”, and also the director: she gradually restructures the play to trace it back to the early reciprocal interactions with significant others, as well as to motivations often in conflict with each other. Unspoken/unspeakable feelings pop up on stage. The strength of this method mainly rests on re-establishing the priority of the embodied social nature of the multifaceted self, eclipsed by the classical talking cure.

Psychoanalytic psychodrama is basically aimed for clinical treatment, but it can provide interesting hints on embodied neuro-cognitive exploration for the following reasons:

– It actualizes embodied theoretical proposals, particularly the ones addressing the bodily self, intersubjectivity, affective quality of verbal and bodily communication, and egocentric/allocentric spatial reference framing, to gradually approach the social-affective realm.
– Psychoanalytic psychodrama puts forward an in-depth investigation of certain concepts, typically neglected by neuroscience, as the ones of internalized moral standards and ideals (Freudian superego) and unconscious conflicts.
– Regarding language, this method puts the accent on social communication, thus on the non-referential aspects of verbal messages. Even more, it emphasizes the “defensive” use of language. This defensive use, aimed at re-establishing a cognitive balance thorough a non-authentic narrative self, is unmasked on stage.
– Through the recursive use of explicit processes of symbolization, psychoanalytic psychodrama handles psychosomatic symptoms by reframing them in a complex affective structure, where to recognize instincts’ sources, aims, and objects (Freud, 1915a).
– From a theoretical and methodological point of view, this technique provides insights into the activation of motor simulation in an ecological context. In recent years, interesting work has been conducted on social cognition. Pioneering research has shown that newborns come into the world wired to socially interact (Castiello et al., 2010) and that is possible to quantify the specific contribution of direct gaze and kinematic information on subjective involvement during interactions (Betti et al., 2018). Moreover, the problem of direct social perception has been reframed in terms of establishing a measurable relationship between movement features and perceived mental states (Becchio et al., 2018). The study of different contexts has highlighted a large increase of self-other integration in divergent-thinking contexts over convergent-thinking ones (Colzato et al., 2016). As far as motor resonance is concerned, a subject of discussion is whether, in some contexts, mirroring the observed action can be disadvantageous, as when we have to perform a joint action. Both physical and social cues affording non-identical complementary actions have been identified (Sartori et al., 2012; Scorolli et al., 2014, 2018; Sacheli et al., 2015; Vesper et al., 2017), but little is yet known about actions and contexts affectively connoted (Bastiaansen et al., 2009; Kuhbandner et al., 2010; Costantini and Ferri, 2013; Lowe et al., 2016; on group membership, see Iani et al., 2011; on motivations for joint actions, see Godman, 2013). Through a specific re-framing or re-adaptation of psychodramatic method, these challenging issues seem feasible to be tackled.
– The acted scene can lead to disentangle the bodily self and the social product of its development, making explicit the individual’s navigation across the internal conflicts. The “social product” of the self-development is affected by the specific environment the individual has had to deal with. The environment may have been more or less responsive to her requests, more or less conflicting with her biological drives and her own personality traits (Scalabrini et al., 2018). The social role is specifically addressed by sociodrama (Moreno, 1934; Rapaggi, 2008), but psychodrama also offers the opportunity to study its impact on the bodily self.
– So far embodied cognition has been mainly focused on investigating the role of sensorimotor system in activating existing repertoires of knowledge: not enough attention has been paid on how body-mind linkages do influence processes of knowledge generation. Some interesting attempts have been fostered by using embodying metaphors to give rise to novel ideas (Leung et al., 2012), or by focusing on measures of creativity assessing both convergent and divergent thinking (Simonton, 2003; Cheng et al., 2008; Kuo and Yeh, 2017). Data converge in suggesting that creativity implicating physical acts does activate processes involved in overcoming mental fixedness, facilitating the psychological process of creative problem solving. Psychoanalytic psychodrama does have lots of potentialities to explore these new perspectives, testing how embodied representations can enlarge existing repertoires of knowledge and/or trigger cognitive processes necessary for generating creative solutions.

To conclude, recent advances in brain connectivity research are providing evidence supporting the convergence of neuroscientific findings and psychoanalysis, emphasizing how this knowledge can impact the “Neuropsychoanalysis” (e.g., Salone et al., 2016). Neuroscientific data often enhance our descriptive knowledge, without substantially improving our understanding of brain functions (Kotchoubey et al., 2016): the atomistic reduction can be overcome through a common effort of both these disciplines. There are excellent premises for a future productive exchange between embodied neuroscientific approaches and psychoanalytic ones: first of all, the common aim should be to integrate the affective and the cognitive aspects of conscious and unconscious mental processes. Mainstream cognitive neuroscience has classically coped with the issue of consciousness focusing on “exteroceptive” objectified forms of consciousness, especially visual consciousness (Solms, 2013, 2014). By considering human subjective experience as the result of higher order cortical processes, the “corticocognitive anthropocentrism” has prevented from considering that having a subjective experience does not necessarily correspond to the fact of being self-aware of such an experience (Alcaro et al., 2017). Both embodied cognition and psychoanalysis recognize and aim to investigate the existence of subjective experiences without self-awareness.

The *cognitive* unconscious and the *psychoanalytic* unconscious still appear to differ. The psychoanalytic (dynamic) unconscious, i.e., a boiling cauldron of impulses and desires, presupposes forces (whose nature is linked to unconscious motivations) that determine the passage of mental contents from the conscious state to the unconscious one, and vice-versa. By cognitive unconscious, instead, it is generally (still) meant the part of mental functioning that is unconscious not because it has been suppressed, but because it has never been known, and therefore, it will never be remembered (neither it would be useful, or therapeutic, to know this part). In the case of the cognitive unconscious, the focus is not on “contents” and emotions, but instead on “processes”. We could also claim that the cognitive unconscious is the part of us that we can never remember, neither forget (Migone, 1995, 2001). Actually, some recent constructs are enlightening on this respect: the “phenomenal minimal self” is defined by Northoff (2013) as the pre-reflexive form of subjectivity that presupposes an experience, defined by certain (pre)conscious qualities, and the implicit sense of being part of such experience. This definition underlines the two necessary and sufficient features of the self: intentionality and conscious sensitivity (Searle, 1991; Alcaro et al., 2017).

For this fruitful dialogue between psychoanalysis and neuroscience to be possible, the theoretical issue to be urgently solved is the agreement on conceptual frameworks through which neuroscientists, psychoanalysts, and philosophers could profitably understand each other. To avoid confusion within the current plethora of psychological constructs (i.e., “core-self,” Panksepp, 1998; “proto-self,” Damasio, 1999; “total self,” Rizzuto, 2008, Gallese, 2009; “phenomenal minimal self,” Northoff, 2013; “minimal self,” Fotopoulou and Tsakiris, 2017; “affective core-self,” Alcaro et al., 2017; “embodied inter-subjectivity,” Fotopoulou and Tsakiris, 2017; “minimal affective subjectivity,” Solms, 2017a), when defining them it could be valuable to systematically refer to the Freudian second topical of the psychic apparatus (id, ego, superego). By this effort, new shared constructs can come up: they may even challenge the founding psychoanalytic ones (e.g., Solms, 2013), however, it is crucial they are systemic concepts, entailing also the human-environment relation (Solms and Panksepp, 2012; Kotchoubey et al., 2016; Solms, 2017a,b).

To deal with the challenge of a mutually productive interchange between neuroscience and psychoanalysis, we also need to carefully examine suitable methodologies and possible novel paradigms. Techniques based on hemodynamic responses, as functional magnetic resonance imaging (fMRI) and positron-emission tomography (PET), have been critical to investigate the impact of emotions on cognitive processes. It has been showed that the amygdala does have a crucial role in the recollection of emotional and motivational memories (fMRI: Dolcos et al., 2005), and that emotional information enhances visual memory recognition (PET: Taylor et al., 1998). Electrophysiological studies (EEG), in spite of the electroencephalography poor spatial resolution, have also provided interesting evidence, such as showing the amygdala neurons’ theta activity (4–8 Hz) during the consolidation of emotional aroused memories (Paré et al., 2002). Nevertheless, the promising technique to be deeply exploited in future research is the functional near-infrared spectroscopy (fNIRS): it allows to investigate cortical responses in face-to-face naturalistic scenarios involving two adult co-actors (Costantini et al., 2013), or infants and their parents (Lloyd-Fox et al., 2015). The advantages of fNIRS encompass non-invasiveness and portability, making this methodology potentially suitable to the psychodramatic setting. Finally, it is noteworthy the current renewed interest for the autonomic nervous system activity: its investigation is feasible through measuring the heart rate variability (extracted from the electrocardiography), the galvanic skin response, and the skin temperature. All these physiological measures, widely used to scrutinize emotions, require fewer sensors (that are often portable), and produce fewer artifacts on respect to EEG. Their use, still suitable to the enacted scene, is particularly promising to investigate the impact of interoception on motivation, emotions, social cognition, and self-awareness.

Beyond linguistic issues and inter-/intra-disciplinary methodological concerns, a framework encompassing embodied proposals and psychoanalytic theorizations should be sketched in a developmental and evolutionary perspective, by integrating philosophical and neuropsychological constructs, and considering ecological connectionist advances. Future interdisciplinary research should embrace action methods, as psychoanalytic psychodrama, since they are suitable candidates to explore the embodiedness and the embeddedness of human beings.

## Author Contributions

CS is the only author and she developed the article structure, wrote the manuscript, and performed the final editing of the text.

### Conflict of Interest Statement

The author declares that the research was conducted in the absence of any commercial or financial relationships that could be construed as a potential conflict of interest.
